# Anatomy-guided dissection plane selection in endoscopic brow lift: a narrative review of supraperiosteal, subperiosteal, and multiplane approaches

**DOI:** 10.3389/fsurg.2026.1856741

**Published:** 2026-07-02

**Authors:** Bo Zhou, Bo Liu, Tianyao Lan

**Affiliations:** 1 Department of Burn and Plastic Surgery, Yichang Central People's Hospital, Yichang, China; 2 The First College of Clinical Medical Science, China Three Gorges University, Yichang, Hubei Province, China; 3 Department of Burn and Plastic Surgery, Beihai People's Hospital, The Ninth Affiliated Hospital of Guangxi Medical University, Beihai, Guangxi Zhuang Autonomous Region, China

**Keywords:** anatomy, endoscope, foreheadplasty, subperiosteal approach, supraperiosteal approach

## Abstract

Endoscopic brow lift has become a widely adopted technique for upper facial rejuvenation due to its minimally invasive nature and favorable aesthetic outcomes. Compared with traditional open approaches, it offers reduced scarring, shorter recovery time, and a lower incidence of sensory complications. Three principal dissection planes are currently employed: the supraperiosteal (subgaleal), subperiosteal, and combined supra–subperiosteal approaches. Each plane differs in anatomical relationships, surgical exposure, biomechanical lifting mechanisms, and risk of neurovascular injury—particularly involving the deep branch of the supraorbital nerve. Recent anatomical and clinical studies suggest that supraorbital nerve tension plays a critical role in limiting brow elevation. Furthermore, considerable anatomical variability exists in the course of the supraorbital nerve, complicating surgical standardization. This review provides a comprehensive comparison of these three approaches, integrating anatomical insights, surgical outcomes, and clinical evidence. Particular emphasis is placed on nerve safety and the rationale for the multiplane approach, which may offer an optimal balance between surgical efficacy and complication avoidance.

## Graphical Abstract

**Figure:**
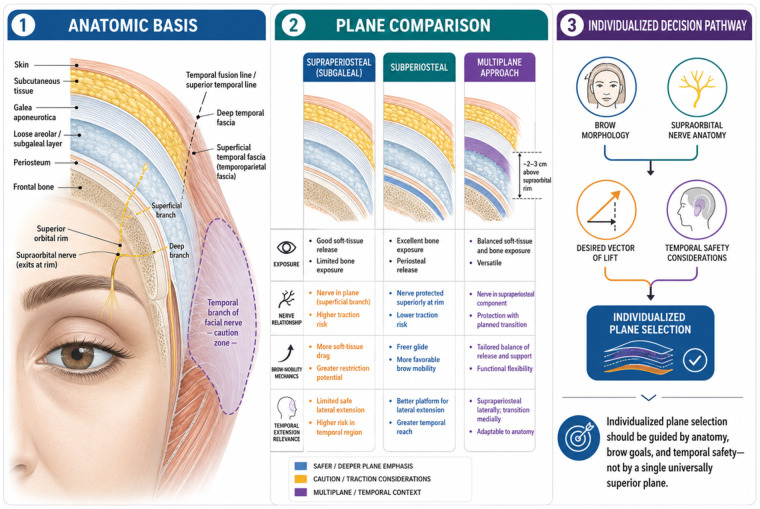
Schematic overview of anatomy-guided dissection plane selection in endoscopic foreheadplasty. Schematic overview of anatomy-guided dissection plane selection in endoscopic foreheadplasty. The left panel illustrates layered forehead anatomy and the course of the supraorbital nerve, particularly its deep branch. The middle panel compares the supraperiosteal, subperiosteal, and combined planes in terms of location, nerve relationship, and operative characteristics. The right panel presents region-specific plane selection, including a multiplane option, as one possible anatomy-guided strategy and does not imply proven superiority of any approach.

## 1 Introduction

In the early 1990s, endoscopic techniques were introduced into facial rejuvenation surgery. Pioneers such as Vasconez (1), Isse (2), and Chajchir (3) were among the first to describe this approach. Compared with traditional open procedures, endoscopic minimally invasive surgery has gained greater patient acceptance due to reduced incision size and less visible scarring (4, 5). In recent years, it has been widely applied in forehead procedures (5–7). Its advantages include shorter recovery time, higher patient acceptance, reduced scalp sensory disturbance, smaller and better-concealed scars, improved safety with fewer complications, and more predictable and durable outcomes (8, 9).

Currently, three principal dissection plane approaches are employed in endoscopic foreheadplasty: the supraperiosteal plane approach, the subperiosteal plane approach, and the combined supra–subperiosteal (multiplane) approach. Vasconez was the first to report endoscopic foreheadplasty using the supraperiosteal approach (9). Subsequently, in 1995, Isse (7) described performing either supraperiosteal or subperiosteal dissection approximately 3 cm above the supraorbital rim, followed by subperiosteal dissection around the neurovascular bundles. This technique initiated ongoing debate regarding the relative merits of these two dissection planes (10).

The central issues in this debate focus on anatomical accessibility, surgical efficacy, and procedural safety between the supraperiosteal and subperiosteal approaches. To integrate the advantages of both techniques, a third approach—the combined supra–subperiosteal (multiplane) dissection—was developed. This method was first described by Rohrich and Beran in 1997 as “deep galeal release” (11).

## 2 Scope statement

This manuscript is intended as a narrative review based on a structured literature search, focusing on anatomy-guided selection of dissection planes in endoscopic brow lift rather than an exhaustive systematic review of all brow-lifting procedures (12, 13). The primary focus of this review is the sensory neuroanatomy of the supraorbital nerve, particularly variations in the course of its deep branch and the relationship of these variations to brow elevation, tissue gliding, and the risk of nerve injury, because these factors directly influence plane selection, tissue release, and fixation strategy (14–17). Because combined-plane and lateral temporal dissection also involve important motor nerve safety considerations, this review also includes a concise discussion of the temporal branch of the facial nerve, temporal fascial planes, and relevant surface and intraoperative landmarks, in order to provide a more complete safety framework for lateral brow-tail elevation (18–22). Accordingly, the purpose of this review is not to claim universal superiority for any single plane, but to synthesize the available anatomical and clinical evidence regarding the relative differences among the supraperiosteal, subperiosteal, and multiplane approaches in terms of safety, mobility, exposure, and clinical indications (13, 14, 17, 23, 24). An evidence-calibrated comparison of the supraperiosteal, subperiosteal, and combined dissection planes is summarized in Table 1.

**Table 1:** Evidence-calibrated comparison of dissection planes in endoscopic brow lift.

Parameter	Supraperiosteal plane	Subperiosteal plane	Combined plane	Key evidence/notes
Dissection level	Between the galea aponeurotica and periosteum (subgaleal/supraperiosteal plane).	Between the periosteum and frontal bone.	Region-specific use of both planes; transition is selected according to anatomy and operative goals rather than a universally fixed distance.	Anatomical and technical descriptions (13–16, 24, 28, 29).
Surgical exposure	Direct access to galeal and muscular soft tissues; may facilitate selective corrugator/procerus treatment and redraping.	Broad exposure of the bony surface and periosteal attachments, with reproducible bony landmarks.	Allows superficial soft-tissue access in one region and bone-level release in another; exposure depends on the selected transition.	Technical and cadaveric evidence (25, 26, 28, 29). Direct comparative clinical data are limited.
Optical cavity	The working space may be narrower and more affected by superficial bleeding; visualization is technique-dependent.	Usually provides a broad, relatively uniform potential space against bone.	Subperiosteal segments may provide a stable cavity, whereas plane transitions add complexity and can interrupt visualization.	Mainly technical/cadaveric observations (25, 28, 29).
Corrugator/procerus access	Often permits direct access to the galeal-muscular layer and selective muscle modification.	Muscle treatment may require additional release or entry into a more superficial layer.	Can be configured to permit targeted muscle access while maintaining a deeper plane elsewhere.	Technical descriptions (26, 29). No high-quality evidence establishes a preferred plane for all patients.
Brow-elevation mechanism	Soft-tissue/galeal redraping relative to the periosteum, followed by fixation.	Release of periosteal attachments and mobilization of the periosteum-soft-tissue unit from bone, followed by fixation or reattachment.	Combines region-specific soft-tissue redraping and periosteal release.	Mechanistic literature (5, 6, 27–32). Mechanism alone does not establish outcome superiority.
Lifting effectiveness	Can achieve clinically useful elevation when release and fixation are adequate.	Can achieve clinically useful elevation through periosteal release and mobilization.	Promising for tailoring release to local anatomy; comparative effectiveness remains uncertain.	A cadaveric comparison found no significant elevation difference between subgaleal and subperiosteal techniques (16, 22, 28). Multiplane reports are noncomparative.
Durability of results	Clinical durability has been reported, but long-term comparative evidence is limited.	Periosteal reattachment provides a biologically plausible fixation mechanism; long-term superiority is unproven.	Intended to combine regional mobility with stable fixation; long-term comparative evidence is limited.	Evidence includes clinical reports and animal/histologic healing studies (23, 29, 33–37). Healing data should not be equated with superior clinical durability.
Recovery profile	Recovery varies with dissection extent, hemostasis, fixation, and patient factors; bruising, edema, or sensory symptoms may occur.	Recovery likewise varies; edema and temporary sensory symptoms may occur.	May increase operative complexity; robust evidence does not show faster or slower recovery than single-plane approaches.	Direct plane-specific comparative data are sparse (23, 29, 33).
Risk of supraorbital nerve injury	Potential for direct contact or transection of the deep branch where it courses in or near the deep galeal layer.	May reduce direct transection because the nerve remains in the flap, but traction or sharp release can cause sensory injury.	May permit avoidance of region-specific vulnerability, but transition requires precise anatomical identification; minimal risk has not been demonstrated.	Risk depends on anatomical variation and technique (13–16, 22, 23, 38, 39).
Relationship to supraorbital nerve	The deep branch may enter or cross the operative plane, with substantial individual variation.	The nerve is generally carried within the elevated soft-tissue flap, although variant branches may approach the periosteum.	Plane choice can be adjusted by region; no single fixed 2–3 cm transition applies to all patients.	Anatomical studies (13–16).
Flap tension	Lower flap tension than subperiosteal dissection was observed in one cadaveric study.	Numerically higher flap tension was observed in the same study; the difference was not statistically significant.	Tension depends on the relative extent of deep release, superficial redraping, and fixation vector; direct comparative data are lacking.	Cadaveric evidence (28); nerve-tension and gliding-layer findings (16).
Perfusion considerations	Superficial dissection may encounter more perforators, but reliable flap vascularity has been reported in technical series.	Major neurovascular structures are generally retained in the flap; clinical proof of superior perfusion is limited.	Perfusion effects depend on the extent and location of each plane.	Available evidence is mainly anatomical or observational (25, 29, 39).
Technical considerations	Requires control of superficial bleeding and careful recognition of nerve branches.	Requires complete periosteal release and avoidance of excessive traction.	Requires accurate plane identification and safe transition between layers; it may have a steeper learning curve.	Technical literature (12, 16, 22, 25, 29). No validated comparative learning-curve data.
Potential clinical use	May be selected when direct soft-tissue or corrugator/procerus management is prioritized.	May be selected when a broad bone-level release and stable optical cavity are prioritized.	May be selected when regional anatomy or different frontal/temporal objectives favor plane switching.	These are anatomy- and goal-based considerations, not evidence-based exclusive indications (12, 16, 22, 25, 29).
Limitations	Narrower working cavity; potential direct nerve contact; visualization may be affected by bleeding.	Less direct superficial muscle access; traction-related nerve risk; tension may increase if release is incomplete or elevation is excessive.	Greater technical complexity; transition landmarks vary; superiority is unproven.	Supported by anatomical, cadaveric, and technical evidence (13–16, 22, 25, 28, 29, 39).
Main uncertainties	Long-term fixation behavior and the clinical significance of direct nerve exposure.	Relationship between periosteal reattachment, fixation strategy, and long-term brow position.	Optimal patient selection and transition strategy; whether plane switching improves outcomes or complications.	Prospective comparative studies are needed (16, 22, 23, 28, 33–37).

Evidence-calibrated comparison of anatomy, operative exposure, biomechanics, and clinical considerations. Because most available evidence is anatomical, cadaveric, technical, retrospective, or derived from animal healing models, the table summarizes trade-offs rather than ranking techniques. Terms such as “optimal,” “minimal risk,” and “more durable” are avoided unless supported by direct comparative evidence.

## 3 Methods

This manuscript was designed as a narrative review based on a structured literature search rather than a strict systematic review (12, 13). PubMed, Scopus, Web of Science, and the Cochrane Library were searched from January 1990 to May 2026. Search terms included “endoscopic brow lift,” “endoscopic forehead lift,” “foreheadplasty,” “supraperiosteal,” “subgaleal,” “subperiosteal,” “multiplane,” “biplanar,” “deep galeal release,” “supraorbital nerve,” “temporal branch of the facial nerve,” “temporal lift,” and “temporal facelift.” The reference lists of key articles were then screened manually to identify additional relevant studies. English-language cadaveric studies, clinical case series, technical articles, and reviews directly relevant to dissection-plane selection, nerve safety, fixation strategy, and temporal extension were preferentially included. Studies focusing exclusively on non-endoscopic techniques and lacking direct relevance to anatomy-guided plane selection were excluded. Given the heterogeneity of the available evidence and the aim of this review to provide anatomical synthesis and clinical interpretation rather than quantitative pooling, no formal risk-of-bias assessment tool or meta-analysis was performed (12, 13, 24).

## 4 Supraperiosteal and subperiosteal plane approaches

Endoscopic brow lift is generally performed through several small scalp incisions, followed by endoscopic-assisted dissection, release of retaining attachments, mobilization of the brow–forehead unit, and fixation in the intended position (24, 25). Depending on the technique and clinical objective, brow depressor muscles may be selectively weakened or released (5, 7, 25). In the supraperiosteal approach, dissection proceeds between the galea aponeurotica and periosteum; in the subperiosteal approach, dissection proceeds between the periosteum and frontal bone.

**Figure 1:**
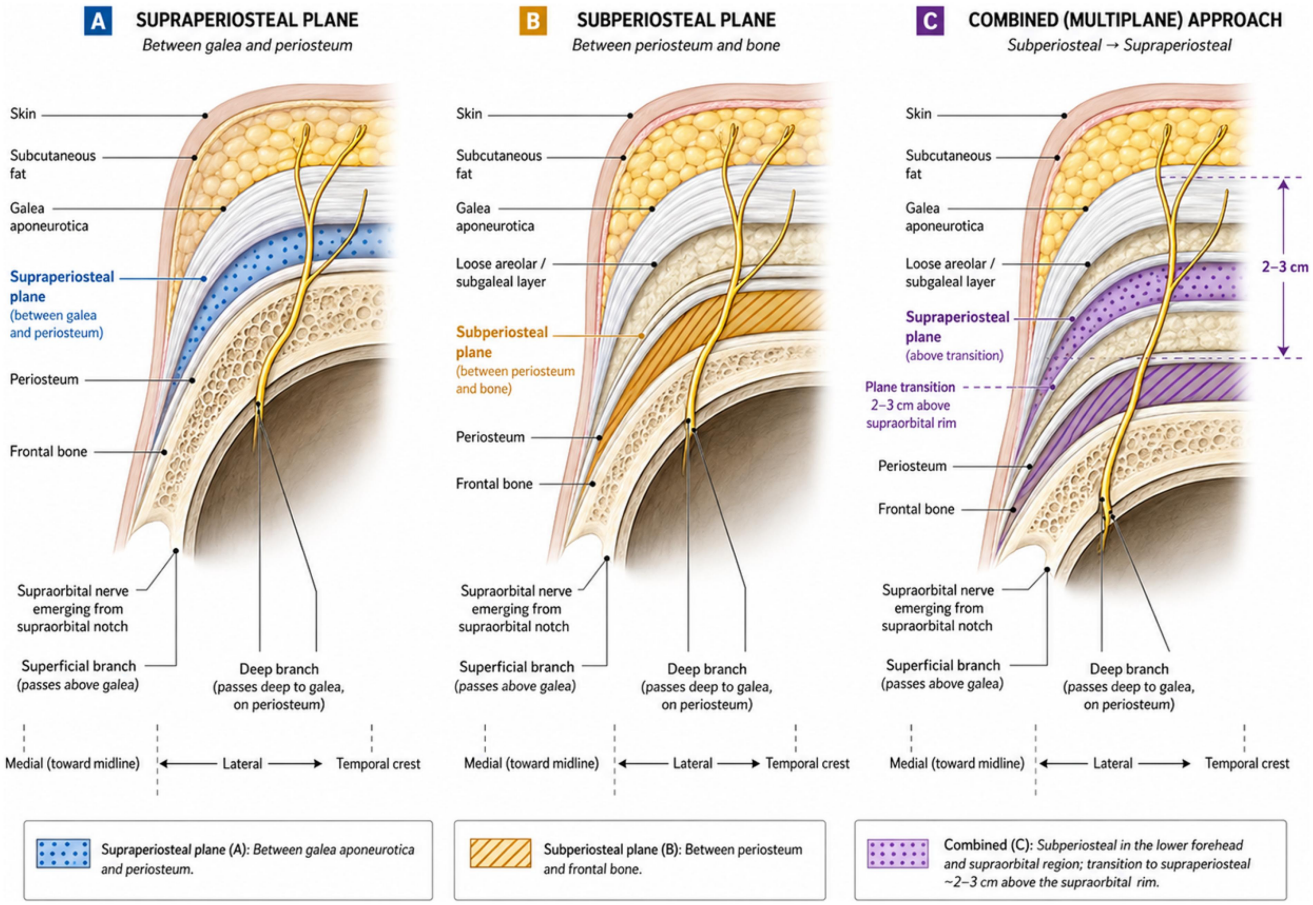
Anatomical illustration of three dissection planes in endoscopic foreheadplasty. Schematic illustration of the three principal dissection strategies. **(A)** Supraperiosteal dissection between the galea aponeurotica and periosteum. **(B)** Subperiosteal dissection between the periosteum and frontal bone. **(C)** Combined supra- and subperiosteal dissection with a region-specific transition between planes. The transition is schematic and should not be interpreted as a fixed universal distance from the supraorbital rim. The anatomical layers and supraorbital nerve are shown to emphasize spatial relationships and plane-specific trade-offs.

**Figure 2:**
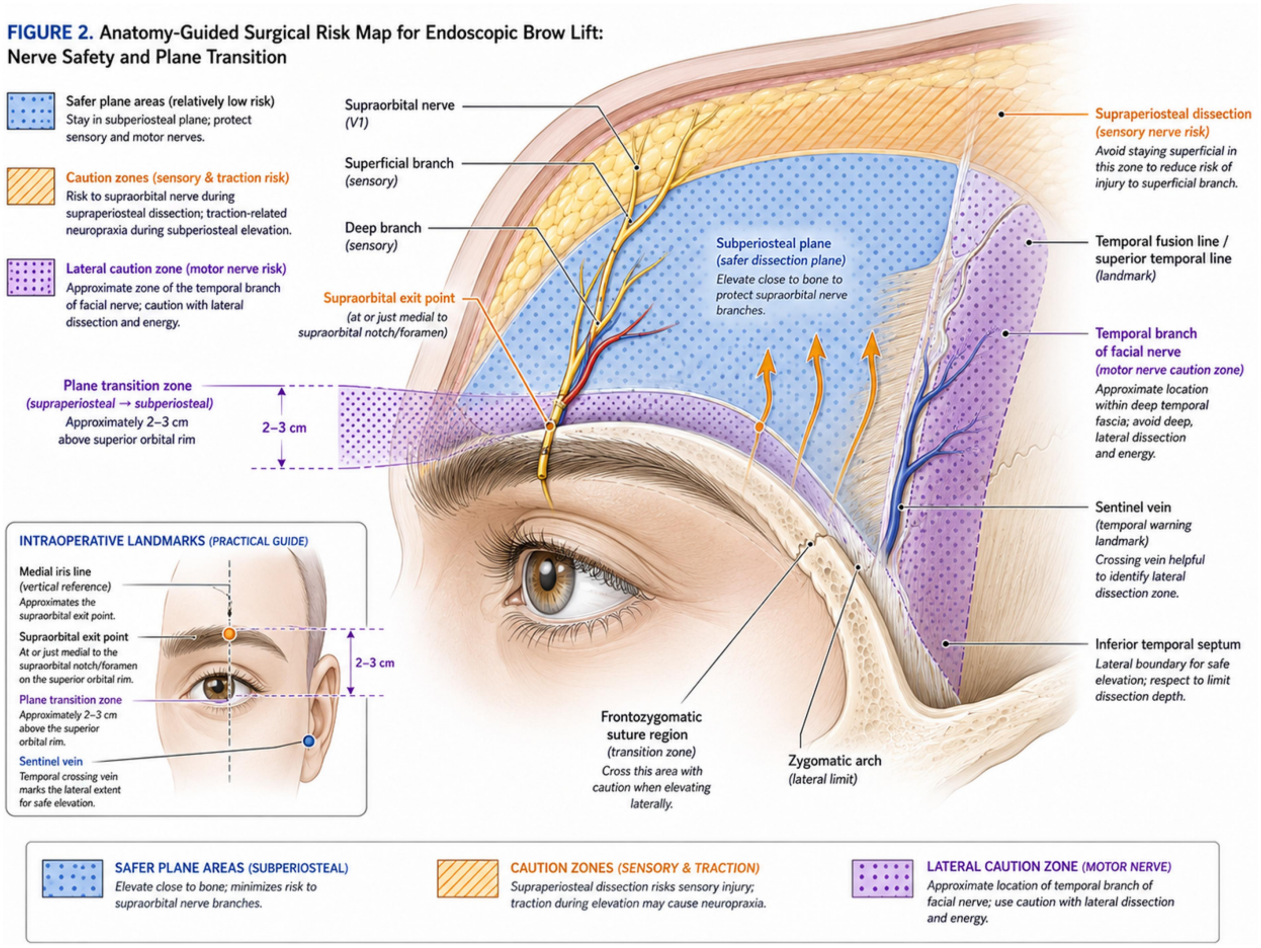
Anatomical-guided surgical risk map for endoscopic brow lift:nerve safety and plane transition. The deep branch of the supraorbital nerve may travel within or close to the galea, placing it near the supraperiosteal plane in some regions and anatomical variants. Subperiosteal dissection generally retains the nerve within the elevated soft-tissue flap and may reduce direct transection, but excessive traction or sharp release may still cause sensory injury. The highlighted regions therefore represent different mechanisms of vulnerability rather than a hierarchy of safety. Plane selection and transition should be individualized according to anatomy and controlled intraoperative dissection.

**Figure 3:**
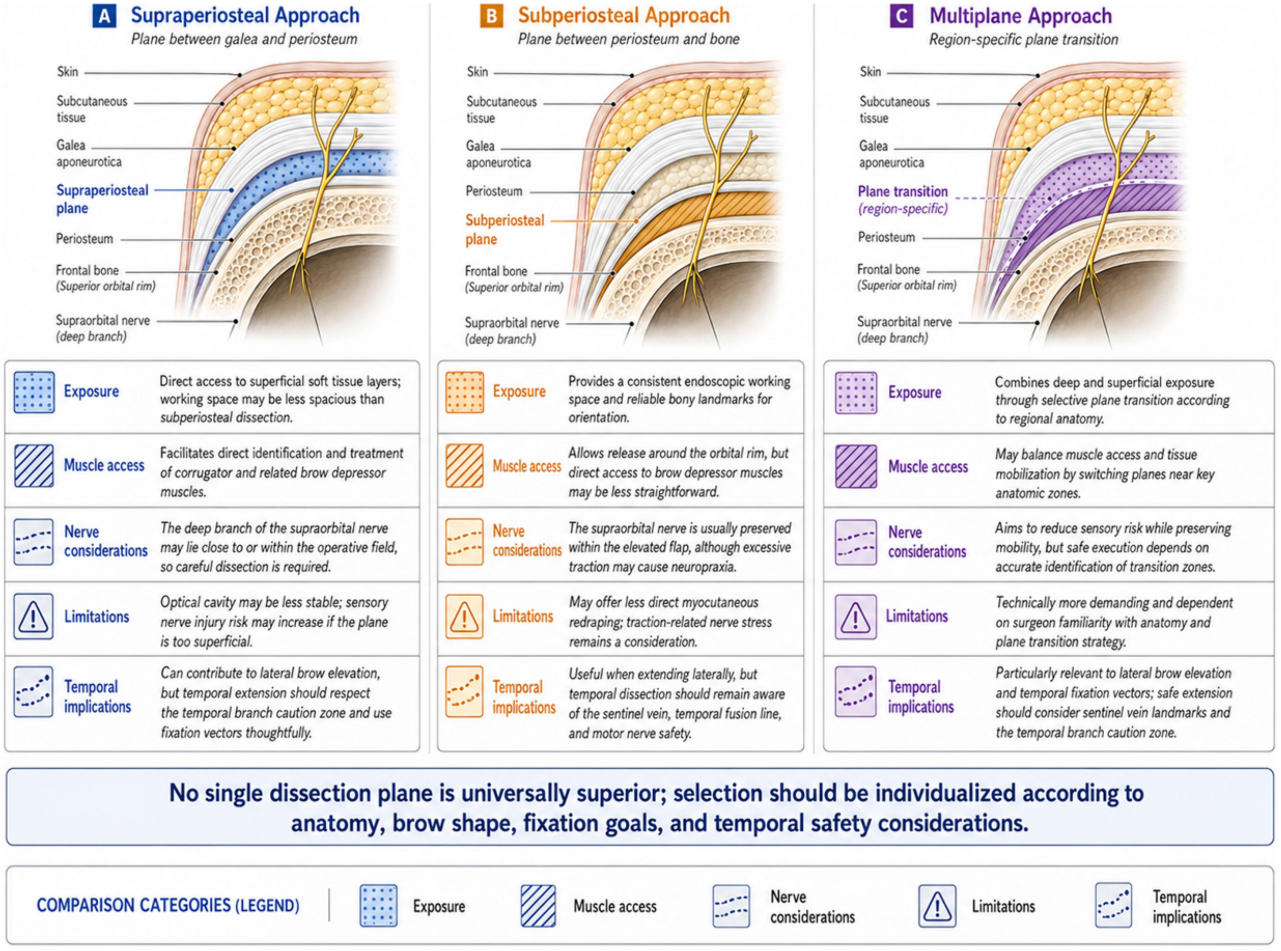
Schematic comparison of advantages and limitations of three surgical approaches. Comparative schematic of commonly described strengths and limitations of supraperiosteal, subperiosteal, and combined dissection. Supraperiosteal dissection may facilitate direct soft-tissue and depressor-muscle management but may place variant supraorbital nerve branches near the operative plane and provide a narrower working cavity. Subperiosteal dissection usually provides a broad bone-level working space and may reduce direct nerve transection, but it offers less direct access to superficial muscle layers and carries traction-related nerve risk. Multiplane dissection permits region-specific plane selection and is anatomically rational in selected cases; however, comparative evidence is insufficient to conclude that it provides superior efficacy or safety. The schematic therefore depicts trade-offs rather than a preferred technique.

From the perspective of anatomical accessibility, the subperiosteal approach offers several advantages. It provides a more consistent and spacious endoscopic working cavity compared with the supraperiosteal plane, and the presence of bony landmarks and fascial attachments facilitates intraoperative orientation, thereby improving procedural efficiency and safety (26). In contrast, the supraperiosteal approach allows easier identification and direct resection of the corrugator supercilii muscles. Moreover, compared with subperiosteal muscle resection, supraperiosteal corrugator resection may be more effective in reducing postoperative abnormal muscle activity (27). In addition, this approach may enhance adherence between the periosteum and galea, thereby improving soft tissue support of the forehead (28). Proponents of the supraperiosteal approach acknowledge that the subperiosteal plane provides a superior optical cavity (26). However, many advocates of supraperiosteal techniques argue that adequate visualization of critical anatomical structures—particularly the corrugator muscles—is of primary importance during endoscopic brow lift (29). Although the subperiosteal plane offers a clearer endoscopic space, most surgeons are able to complete dissection of the upper two-thirds of the forehead without endoscopic assistance (6, 26). Furthermore, some authors suggest that the supraperiosteal plane facilitates access to the deep portions of the procerus and corrugator muscles, allowing more refined and limited muscle modification (30). They also argue that concerns regarding an “insufficient optical cavity” in the supraperiosteal plane are overstated, as the reflective surface of the periosteum can enhance illumination and provide visualization comparable to that of the subperiosteal plane. Taken together, both supraperiosteal and subperiosteal approaches present distinct advantages, and no definitive conclusion can be drawn regarding superiority in terms of anatomical convenience. The anatomical relationships among these planes, their nerve-safety considerations, and their plane-specific trade-offs are schematically illustrated in Figures 1–3.

With respect to lifting efficacy, many proponents of subperiosteal techniques consider the periosteum to be a key limiting structure for brow elevation, and therefore emphasize the importance of periosteal release (6, 7, 31, 32). It has been reported that periosteal release at the supraorbital rim may achieve approximately 4–10 mm of brow elevation (31, 32). In contrast, advocates of the supraperiosteal approach emphasize the underlying etiology of brow ptosis. They argue that decreased muscle tone and loss of skin elasticity are the primary contributors to brow descent, whereas the periosteum retains its tensile strength over time and does not undergo significant displacement (30). Some studies have described age-related brow ptosis as a phenomenon in which the skin and muscle descend relative to a relatively fixed periosteum (referred to as a “creep phenomenon”) (33). Based on this concept, elevation of the subgaleal soft tissue layer relative to the periosteum provides a logical mechanism for rejuvenation of the upper third of the face (30). Accordingly, some authors suggest that the supraperiosteal approach may yield superior rejuvenation outcomes. However, cadaveric studies have demonstrated no significant difference in brow elevation between supraperiosteal and subperiosteal approaches under both resting conditions and standardized traction forces (2.2 kg) (29). Regarding durability and recovery, comparative studies have yielded conflicting results. Troilius (34) proposed that the subperiosteal approach may provide more durable results due to the firm adhesion between the non-elastic periosteum and bone, compared with the more elastic attachment between the galea and periosteum in the supraperiosteal approach. The healing process between bone and periosteum has been reported to range from 7 days to 6–12 weeks (35–38). Conversely, other studies suggest that adhesion between the galea and periosteum occurs more rapidly than periosteal reattachment to bone, representing a potential advantage of the supraperiosteal approach (30). Overall, no definitive conclusion has been reached regarding superiority in terms of lifting efficacy, durability, or recovery.

From a safety perspective, injury to the deep branch of the supraorbital nerve remains a major concern. Damage to this nerve can result in permanent sensory disturbance or numbness of the vertex scalp (15, 39). Some studies have suggested that the supraperiosteal approach carries a higher risk of injury to the deep branch of the supraorbital nerve (40). Knize (14) demonstrated that the deep branch exits the periosteal surface, traverses the deep layer of the galea aponeurotica, and courses within the galea toward the vertex, providing sensory innervation to the scalp. Consequently, dissection in the supraperiosteal plane may risk transection of this branch. In contrast, during subperiosteal dissection, the deep branch is typically preserved within the elevated soft tissue flap, reducing the likelihood of direct injury. Based on this anatomical relationship, Knize proposed that the subperiosteal plane may represent a safer dissection plane, although detailed anatomical variability was not fully characterized. However, other studies have indicated that excessive traction during subperiosteal dissection—particularly when attempting to achieve greater lifting—may also result in nerve injury (17, 23). The ongoing controversy regarding plane selection is largely attributable to incomplete understanding of the anatomical course of the deep branch of the supraorbital nerve. From another perspective, some authors suggest that the supraperiosteal plane allows easier direct visualization of neurovascular bundles (30). In contrast, during subperiosteal dissection, sharp division of perpendicular connective tissue bands (referred to as “periosteal–galeal adhesion bands”) may increase the risk of inadvertent injury to neurovascular structures. Regarding flap vascularity and tension, some studies suggest that the subperiosteal approach better preserves vascular supply (26). Conversely, others have proposed that the supraperiosteal approach may compromise blood supply and contribute to complications such as incision-site alopecia (40). However, clinical observations indicate that the supraperiosteal plane generally maintains reliable vascularity (30). In terms of flap tension, one study found that median flap tension was higher in the subperiosteal approach compared with the supraperiosteal approach, although the difference was not statistically significant (*P* > 0.05) (29). Therefore, with regard to neurovascular injury, flap perfusion, and tissue tension, no consensus has been reached, and neither approach demonstrates clear superiority in clinical practice.

## 5 Combined subperiosteal and supraperiosteal plane

To integrate the advantages of both dissection planes, a third approach—the combined supra–subperiosteal (multiplane) approach—has been developed. This technique was first described by Rohrich and Beran in 1997 as “deep galeal release” (11). It involves initial dissection in the subperiosteal plane extending to approximately 2 cm above the supraorbital rim, followed by transition to the supraperiosteal plane. Kim (17) proposed that the combined supra–subperiosteal approach represents the optimal surgical plane. He suggested that supraperiosteal dissection offers the advantage of reduced suspension-related traction stress on the supraorbital nerve. However, he also observed that in the superolateral and mid-lateral forehead regions, the deep branch of the supraorbital nerve may traverse the periosteal layer. Therefore, to protect all sensory nerve branches, he recommended performing subperiosteal dissection in the superolateral and mid-lateral forehead, while adopting the supraperiosteal plane in the inferior forehead and brow region. Multiple authors have advocated the use of a multiplane approach in endoscopic brow lift (11, 17, 41–44). Fogli further elaborated on the advantages of this technique, emphasizing its ability to optimize surgical exposure while preserving neurovascular structures (41). This plane-switching strategy combines the strengths of both supraperiosteal and subperiosteal approaches while minimizing their respective limitations, and it has now been widely adopted in endoscopic foreheadplasty. Yi (44) reported in 2011 that the use of this technique significantly reduced the incidence of postoperative sensory disturbances.

Intraoperatively, the transition point between the supra- and subperiosteal planes should not be determined mechanically by a fixed distance of “2–3 cm above the supraorbital rim” alone, because the exit pattern, branching configuration, and fascial relationship of the superficial and deep branches of the supraorbital nerve vary substantially among individuals (15–17). A safer strategy is to combine multiple landmarks, including preoperative marking of the supraorbital notch/foramen and its surface projection, intraoperative recognition of fascial-layer changes in the forehead, the anatomic transition near the superior temporal line/temporal fusion line, and treatment of the medial zygomaticotemporal vein/sentinel vein region as a temporal caution zone (15, 16, 19, 20). If more lateral temporal extension is performed, available anatomical studies suggest that dissection should gradually shift to a more superficial plane within the caution zone approximately 3 cm superolateral to the lateral canthus, in order to reduce the risk of injury to the temporal branch of the facial nerve within the interval between the superficial and deep temporal fascia (19, 21). When needed, transillumination or Doppler-assisted localization of superficial venous/vascular landmarks may be used as an adjunct, but direct validation of these techniques in the endoscopic brow-lift literature remains limited; they should therefore be regarded as auxiliary tools rather than substitutes for anatomical knowledge and controlled dissection (evidence limited) (13, 19–21).

Taken together, these data suggest that both supraperiosteal and subperiosteal approaches can achieve comparable lifting outcomes when appropriately applied, while excessive traction or inadequate understanding of nerve anatomy may increase the risk of complications (13, 16).

## 6 Temporal-region

The temporal component deserves separate consideration because the position of the lateral brow tail is determined not only by frontal release, but also by temporal fascial planes, fixation vectors, and the mobility of the upper-midface soft tissues (17, 23, 45, 46). Anatomically, the temporoparietal fascia is continuous with the galea and the superficial musculoaponeurotic system (SMAS), whereas the temporal branch of the facial nerve enters the temporal region after crossing the zygomatic arch and is often located within the fascial interval between the superficial and deep temporal fascia; therefore, the safety of lateral dissection cannot be inferred from supraorbital nerve anatomy alone (18, 19, 22, 45). Classical surface landmarks such as Pitanguy's line remain useful for initial orientation, but contemporary anatomical studies suggest that the course of the temporal branch should be understood more safely as an individually variable risk zone rather than a fixed and absolute line (19, 21, 22).

In addition, the medial zygomaticotemporal vein, clinically referred to as the sentinel vein, may serve as an important warning landmark in the lateral periorbital and temporal region because of its proximity to the temporal branch of the facial nerve and its value for orientation during temporal endoscopic or direct brow-lifting procedures (19, 20). From a biomechanical perspective, the temporal fixation vector affects not only the degree of brow elevation but also brow-tail shape and recruitment of the upper midface. Recent temporal facelift literature suggests that when temporal dissection is conceptualized as part of an upper-face-to-midface suspension continuum, it may contribute to lateral brow-tail elevation, lateral canthal support, and a degree of midface lifting; however, such evidence is mainly derived from technical series and retrospective cohorts and is insufficient to establish definitive superiority over conventional endoscopic brow-lift dissection planes (evidence limited) (17, 23, 24, 45, 46).

## 7 Summary and outlook

The choice of dissection plane is critical to the success of endoscopic foreheadplasty. Both the supraperiosteal and subperiosteal approaches have distinct advantages and limitations. The supraperiosteal approach facilitates release of the forehead flap and allows easier identification and direct resection of the corrugator supercilii muscles (28), thereby effectively reducing forehead rhytides. In addition, this plane is relatively clean and associated with rapid recovery (30). However, it may increase the risk of injury to the supraorbital and supratrochlear nerves (40), and it generally provides a less optimal endoscopic working space compared with the subperiosteal plane (29). In contrast, the subperiosteal approach offers a more favorable and stable endoscopic optical cavity (29), provides protection to the aforementioned neurovascular structures, and allows effective hemostasis (14, 26). It is also considered to provide more durable lifting outcomes (28). However, its effectiveness in improving forehead wrinkles—particularly in terms of corrugator muscle management—is relatively limited (23). This limitation is likely due to the firm and inelastic nature of the periosteum, which restricts the mobility of the forehead flap during pure subperiosteal dissection. With regard to supraorbital nerve injury, both approaches carry inherent risks. The supraperiosteal approach may predispose to nerve injury due to its anatomical proximity to the neurovascular structures (14), whereas excessive traction in the subperiosteal plane, particularly when attempting to achieve greater lifting, may also result in nerve damage (17, 23). When used independently, each approach offers specific advantages but is also associated with certain limitations. The ongoing controversy regarding the optimal dissection plane is largely attributable to incomplete understanding of the anatomical course of the deep branch of the supraorbital nerve.

Current evidence is insufficient to establish definitive superiority of any single dissection plane in endoscopic brow lift (17, 23, 24, 29). A more balanced conclusion is that different planes provide different trade-offs among surgical exposure, forehead tissue gliding, depressor-muscle management, fixation behavior, and neurovascular risk (14–17, 23, 29). Therefore, the multiplane approach should be described as an anatomically rational and clinically relevant option rather than as a universally superior standard solution (17, 23). In particular, when variations in the deep branch of the supraorbital nerve, the mobility of the frontotemporal gliding layer, and motor nerve safety in the lateral temporal region are considered, region-specific plane transition has clear theoretical appeal; however, its evidence base currently derives mainly from anatomical studies, technical descriptions, and retrospective series (evidence limited) (16, 17, 19, 23, 45, 46). Future studies should focus on more refined classification of supraorbital nerve branching patterns, quantitative description of temporal risk zones and fascial transition areas, objective correlation between fixation vectors and brow-shape changes, and prospective comparisons of long-term outcomes among different dissection planes (16, 17, 19, 20, 24, 46).
